# Decoupling Health and Negative Affect in Late Adulthood: Self-Perceptions of Aging as a Psychological Buffer

**DOI:** 10.1093/geroni/igaf122.4093

**Published:** 2025-12-31

**Authors:** Han-yun Tseng, Manfred Diehl, Stephen Aichele, Hans-Werner Wahl, Oliver Schilling

**Affiliations:** University of Colorado, Aurora, Colorado, United States; Colorado State University, Fort Collins, Colorado, United States; Colorado State University, Fort Collins, Colorado, United States; Heidelberg University, Heidelberg, Baden-Wurttemberg, Germany; Heidelberg University, Germany, Heidelberg, Baden-Wurttemberg, Germany

## Abstract

**Objectives:**

This study revisited the paradox of well-being in a 20-year longitudinal dataset, testing whether self-perceptions of aging (SPA), a modifiable psychological resource, buffer the impact of declining health on emotional well-being in midlife and later adulthood.

**Methods:**

Data were drawn from the Interdisciplinary Longitudinal Study of Adult Development and Aging (ILSE), which followed participants from age 60 to 80 (C30 cohort) and age 40 to 60 years (C50 cohort). Negative affect was measured using four longitudinally invariant items from the Zung Depression Scale; health was objectively assessed by physicians; SPA was measured by the Attitudes Toward Own Aging scale (ATOA). Latent growth curve models tested longitudinal associations and SPA moderation.

**Results:**

Health declined significantly in both cohorts, whereas negative affect remained stable, consistent with the paradox of well-being. SPA significantly moderated the health-affect relationship, but only in the older cohort (C30 cohort). Specifically, in older individuals with more positive SPA, the association between health decline and negative affect was significantly weaker compared to those with more negative SPA. No moderation effect of SPA on health-affect associations emerged in the younger cohort (C50 cohort). The findings held after controlling for a range of sociodemographic and personality characteristics.

**Conclusions:**

These findings support the proposition that SPA influence emotional resilience in later life. Additionally, the paradox of well-being may be contingent on SPA, such that positive views of aging help preserve emotional well-being despite physical decline. This finding points to the potential of SPA-focused psychological interventions to promote well-being in aging populations.

